# Molecular testing for adult type Alport syndrome

**DOI:** 10.1186/1471-2369-10-38

**Published:** 2009-11-17

**Authors:** Genevieve Pont-Kingdon, Kelli Sumner, Friederike Gedge, Chris Miller, Joyce Denison, Martin Gregory, Elaine Lyon

**Affiliations:** 1 ARUP institute for Clinical and Experimental Pathology, Salt Lake City, UT, USA; 2 ARUP Laboratories, Salt Lake City, UT, USA; 3 University of Utah, School of Medicine, Salt Lake City, UT, USA

## Abstract

**Background:**

Alport syndrome (AS) is a progressive renal disease with cochlear and ocular involvement. The majority of AS cases are X-linked (XLAS) and due to mutations in the COL4A5 gene. Although the disease may appear early in life and progress to end stage renal disease (ESRD) in young adults, in other families ESRD occurs in middle age. Few of the more than four hundred mutations described in COL4A5 are associated with adult type XLAS, but the families may be very large.

**Methods:**

We classified adult type AS mutation by prevalence in the US and we developed a molecular assay using a set of hybridization probes that identify the three most common adult type XLAS mutations; C1564S, L1649R, and R1677Q.

**Results:**

The test was validated on samples previously determined to contain one or none of these mutations. In the US, the test's clinical specificity and sensitivity are estimated to be higher than 99% and 75% respectively. Analytical specificity and sensitivity are above 99%.

**Conclusion:**

This test may be useful for presymptomatic and carrier testing in families with one of the mutations and in the diagnosis of unexplained hematuria or chronic kidney disease.

## Background

Alport syndrome (AS) is a progressive inherited disease characterized by persistent hematuria that progresses to ESRD. The disease is often accompanied by progressive hearing loss and ocular lesions. AS is due to defects in type IV collagen alpha chain 3, 4 or 5 encoded respectively by the COL4A3, COL4A4 and COL4A5 genes. The majority (>80%) of AS is X-linked with mutations found in the COL4A5 gene [1]. Autosomal recessive and dominant forms also exist due to mutations in COL4A3 or COL4A4.

More than 400 mutations causing XLAS have been described in COL4A5 http://www.arup.utah.edu/database/ALPORT/ALPORT_welcome.php. Genotype-phenotype correlations have been established by several groups [2-4]. Large rearrangements, nonsense and frameshift mutations cause juvenile onset disease in most patients. Other mutations such as some splice variants and glycine substitutions in the GXY collagen repeats, specifically in exons 1-20, confer later onset disease [2]. Amino acid substitutions in the N non-collagenous (NC1) domain of the alpha 5 chain were described in families with adult onset (ESRD at >30 years old) XLAS with or without hearing loss [5-7].

Among mutations for adult type AS, L1649R was discovered in 9 kindreds and traced to a single common ancestor in New England more than 200 years ago [6]. C1564S was discovered in one very large kindred residing in the US [1]. R1677Q was found in three families of Ashkenazi Jewish descent [5]. Phenotypes associated with three mutations (C1564S, L1649R and R1677Q) are distinct with the effect of C1564S being more severe than L1649R or R1677Q.

In this manuscript we address the prevalence of these mutations, in the US among other mutations responsible for adult type AS and we describe a cost effective and rapid molecular test targeted to these 3 mutations.

## Methods

Adult-type AS was defined as median age of ESRD greater than 30 years in males. Cases were identified from the Utah Alport Study. The median age of ESRD in affected males in kindreds with a known COL4A5 mutation was calculated by Kaplan-Meier survival analysis (SPSS 16.0 for Mac). For two mutations, no males had reached ESRD. In these kindreds the median age of ESRD was expressed as greater than the age of the oldest living affected male. Gene-carrying males were stratified by mutation independently of whether they came from one family or from several families with the same mutation.

All gene-carriers known by June 2009 were tabulated and the proportions attributable to each mutation were calculated. Males were designated as affected (and therefore gene-carriers) if a mutation had been shown in the individual by genetic analysis using any of several methods [1,5,6,8], or if a mutation had been shown in the kindred and the individual was on a line of descent for X-linked inheritance and had hematuria or renal failure. Females were designated as gene-carriers if a mutation had been shown in the individual by genetic analysis or if a mutation had been shown in the kindred and the individual was on the line of descent for X-linked inheritance and had hematuria, renal failure or was an obligate carrier.

For the molecular assay, DNA, obtained from approved IRB protocols and described previously [5,6], was used for validation. Positive samples comprised both males and females. Nine had the C1564S mutation, 12 the R1677Q mutation and 13 the L1649R mutation. Samples with mutations in other COL4A5 exons and one non-affected male sample were used as confirmed negatives for the targeted mutations. Twenty of these were tested for C1564S and R1677Q and 17 were tested for L1649R.

Exons 49 (C1564S), 50 (L1649R) and 51 (R1677Q) were amplified by PCR in a LightCycler instrument (Roche Applied Science) using 0.5 μM of primers and 0.2 μM of probes shown in Table 1, 3 mM MgCl2 1× LightCycler DNA Master HybProbe (Roche Applied Science), UNG (0.1 unit of AmpErase Uracil N-glycosylase). For each mutation, probes comprised an anchor probe labeled in 3' with FITC and a reference probe that matches the non -mutated sequence, labeled in 5' with either LC705 (C1564S and L1649R) or LC640 (R1677Q) and blocked in 3' with a C3 blocker (Idaho Technology, Salt Lake City, Utah). Exons 49 and 51 were multiplexed while exon 50 was amplified individually with the following protocol: After pre-warming the instrument at 94°C for 10 min. and cooling to 30°C for 1 min., one UNG decontamination step was performed for 3 sec. at 50°C followed by UNG enzyme inactivation at 95°C for 5 min. Amplification was for 60 cycles of 95°C for 0 sec, 60°C for 10 sec and 75°C for 15 sec. Ramp rate (°C/sec) between the steps were respectively 20, 20 and 2. After amplification, the PCR product was melted as follow; 94°C for 30 seconds followed by cooling and holding at 45°C for 2 minutes. Temperature was then increased to 85°C at a rate of 0.1°C/sec while fluorescence was recorded in both the F2 (LC640) and the F3 (LC705) channels of the instrument in presence of the color compensation file. Negative derivative of melting curves were calculated using the LCDA 3.5 software and *T*_m _of the probes was determined.

**Table 1:** primers and probes sequences for each mutation

C1564S	
Forward primer:	5' gaaggcttccaatgaagcag
Reverse primer:	5' tgatgacaaatgcaaggaaga
Anchor probe:	5' ctgactgtgaactgcgatcaccacagctggagctt-FITC
Reference probe:	5'LC705-acatactgcaCatctggtaaagg-C3 block
L1649R	
Forward primer:	5' tttcgttcagctcccttc
Reverse primer:	5' tataagcactttacctgaacatgtctg
Anchor probe:	5' ggtacctgtaactactatgccaactcctaca-FITC
Reference probe:	5'LC705-cttttggcTggcaactgt-C3 block

R1677Q	
Forward primer:	5' ccagaaaatgtggatctgattg
Reverse primer:	5' ttggggacaatgagacactg
Anchor probe:	5' cacaaaaggaattcttcaaaatgttatgtcctcttcat-FITC
Reference probe:	5'LC640-cacacttgacatCggctaattc-C3 block

## Results and Discussion

### Mutations at 3 loci account for the majority of all cases of adult-type AS in the US

Mutations in Col4A5 were determined in several hundred adult type XLAS known or inferred gene -carriers (Table 2). Twenty two mutations were determined among which 9 were previously reported but not analysed for phenotype. L1649R mutation is found in 48.6% of all gene-carriers for adult-type AS, the C1564S mutation in 17.4% and R1677Q mutation in 7.5%. When C1564R and R1677P are included, missense mutations at positions 1564, 1649, and 1677 account for 76.4% of all mutant genes for adult-type AS in our kindreds.

**Table 2:** Median age of ESRD in affected males and numbers of known gene-carriers in adult type Alport syndrome

COL4A5 mutation [ref of previously published mutation]	ESRD age	Male	Female	Total	% of total
leu1649arg [6]	39	150	222	372	48.6

cys1564Ser [1]	32	52	81	133	17.4

arg1677gln [5]	50	19	38	57	7.5

gly1170ser	38	13	11	24	3.1

c.2476delC	44	11	12	23	3.0

gln1234ter	42	7	16	23	3.0

c.1424-20 T>A	32	7	12	19	2.5

cys1564arg	33	9	9	18	2.4

pro1584 leu	34.5	6	9	15	2.0

arg1563gln [2]	34.5	5	8	13	1.7

gly1030ser [10]	38	9	3	12	1.6

c.687+1G>A	33	7	4	11	1.4

gly763glu	42	5	3	8	1.0

gly548asp	45	6	2	8	1.0

gly1244asp [8]	57	3	3	6	0.8

arg1677pro [8]	37.5	2	2	4	0.5

gly295asp [8]	49.5	3	1	4	0.5

gly719arg	>38	2	2	4	0.5

c.3554 -3C>G [10]	31	2	2	4	0.5

gly533glu	32	1	2	3	0.4

c.4177delC	35	2	0	2	0.3

gly325gln	>41	1	1	2	0.3

Total		322	443	765	100.0

Information in the Utah Alport Study has been collected since 1949 when Tyler and Perkoff began to investigate nephritis in four related men who were subsequently shown to carry the C1564S mutation [9]. There is obvious potential for ascertainment bias favouring larger numbers in this kindred because the C1564S family was the first to be studied and there has been more time to ascertain members than in other kindreds. We cannot completely exclude ascertainment bias, but during the 60 years of the Utah Alport Study cases have accumulated from across the whole of the United States and for most of this time our center was the only one collecting cases on a systematic basis. There has been ample opportunity to discover a large proportion of the kindreds with AS in the US. As an example, 22 apparently unrelated families with the L1649R mutation have come to light and this is now the most prevalent mutation. Twelve of these families were shown by haplotype analysis to have arisen from a common ancestor [6]. Although it is possible that other large kindreds exist but have not come to light over 60 years of data collection, this seems unlikely. It is also possible that there are as yet unrecognized branches of known families.

Ascertainment bias within kindreds is shown in the ratio of females to males identified. For an X-linked condition at equilibrium there will be twice as many female as male gene-carriers. The overall F:M proportion in our study was 1.39. It was higher in the 3 largest kindreds at 1.48, 1.56, and 2.0 respectively, but only 0.77 in the families with 4 or fewer identified gene-carriers. Gender-specific ascertainment bias is expected in an X-linked condition because males are more severely affected and thus come to notice.

Despite inaccuracies introduced by ascertainment bias, our data are the only systematic compilation of adult-type AS mutations in the US. We believe that they provide a reasonable approximation of the relative frequencies of different adult-type mutations.

### Molecular assay for the detection of L1649R, R1677Q and C1564S mutations

**Figure 1:**
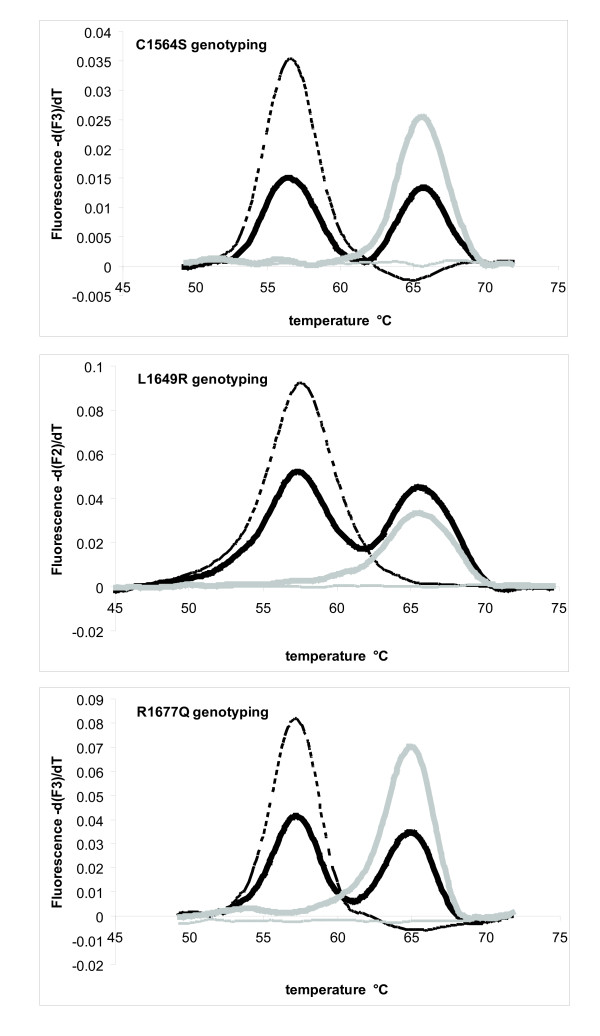
**Genotyping of C1564S, L1649R, and R1677Q by melting curve analysis**. Each graph analyzes one of the mutations with DNA from a positive male (dotted line), a female carrier (black line), a normal individual (grey line) and a no template control (thin grey line). Fluorescence on the Y axis is the negative derivative of fluorescence in function of temperature. Positions of the peaks indicate the dissociation of the probes with a given allele. As probes are homologous to the normal allele, lower *T*_m_s correspond to the mutant alleles and higher *T*_m_s to the normal allele.

We have developed a molecular assay using fluorescently labeled hybridization probes that detect the three main US mutations. The C1638Y mutation recently described in New Zealand [7] and other rare mutation (Table 2) are not included in this test. The assay uses melting curve analysis of three allele specific hybridization probes. Melting analysis of the C1564S probe (Figure 1) shows one single melting curve in the four positive males with a melting temperature (*T*_m_) of 56.7°C, two melting curves in the five heterozygotes females (*T*_m_s = 56.7°C and 65.8°C), and a single melting curve in 22 samples negative for the mutation (*T*_m _= 65.8°C). The curves correspond to the dissociation of the probe with the mutant and normal allele, respectively. Genotyping of this locus was 100% accurate.

The L1649R probe was tested on nine positive males, four positive females and 17 samples with other mutations in COL4A5. Melting analysis of the probe revealed a single peak (*T*_m _= 57.4°C) in positive males, two peaks in heterozygous females (*T*_m_s = 57.4°C and 65.3°C) and a single peak in samples without the mutation (*T*_m_s = 65.3°C). Accuracy was 100%.

The R1677Q mutation was analyzed in six positive males, five carrier females and a positive sex-undetermined sample. Twenty-two samples without the mutation were also tested. Two of these had mutations in the same codon as R1677Q (R1677X [10] and R1677P [8]) and are associated with juvenile XLAS (unpublished). The melting peak corresponding to the presence of the mutation is 57.4°C while the melting peak of the normal allele is 65.1°C. The R1677X mutation was detected by a melting curve at 60.0°C and the R1677P mutation at 56.3°C, both distinguishable from the targeted mutation. All positives and negative samples were correctly identified by the assay.

Precision of the assay was performed by analyzing a sample of each genotype in triplicate in one experiment (within run variation) and in five independent experiments (between run variation). Values of *T*_m_s and difference of *T*_m_s between the curves (in heterozygous females) were used to determine the precision of the assays. Two standard deviations were calculated from the averages and did not exceed 0.5°C for *T*_m_s and 0.25°C for delta *T*_m_s.

The assay identified all the targeted mutations present in the positive samples and none in negative samples; it has accuracy, analytical sensitivity and specificity above 99%. Because the mutations are familial and are carried on single haplotypes, it is unlikely that unreported polymorphisms under the PCR primers or the probes will affect the analytical sensitivity or specificity of the assay.

Males with one COL4A5 mutation are predicted to be affected with AS while women with one mutation are carriers and may or may not develop symptoms. The C1564S, which likely disrupts the collagen IV network by disabling formation of a disulfide bond is the most severe of the three. Fifty percent of males positive for C1564S are expected to have ESRD by age 32 and hearing loss by age 25. The mutation L1649R substitutes a neutral amino acid for a charged amino acid in the NC1 domain. ESRD is expected for 50% of L1649R positive males by age 38 and hearing loss by age 45 [6]. Typical age of ESRD in males with the R1677Q mutation is 38 years [5].

Other mutations associated with adult type AS, Table 2 and [2,7] or with juvenile type AS will not be detected. Clinical sensitivity for adult type XLAS in U.S. Caucasian population is estimated to be around 75% and clinical specificity is 99%. Other testing approaches such as sequencing and detection of duplications and deletions in COL4A5, COL4A3 and COL4A4 need to be used to detect all genetic mutations implicated in AS.

## Conclusion

This assay is designed to detect mutations responsible for the majority of adult type AS in the US. It is recommended for diagnostic, pre-symptomatic, or carrier testing of individuals from families known to carry one of the COL4A5 mutations tested: C1564S, L1649R or R1677Q. Adult type AS is likely substantially under-diagnosed because the X-linked pattern of inheritance frequently conceals the familial nature of the disease and because hearing loss is subtle in many cases. This assay may help elucidate otherwise unexplained hematuria or chronic kidney disease in men, and unexplained hematuria in woman with a family history of chronic kidney disease.

## Competing interests

The authors declare that they have no competing interests.

## Authors' contributions

GPK designed the assay, acquired and analyzed data. KS performed experiments. FG sequenced the samples with the mutations. CM was involved in test interpretation and utility, JD collected pedigree information and MG provided genotyped samples and performed the demographic analyses. EL directed the project. All authors read and approved the final manuscript.

## Pre-publication history

The pre-publication history for this paper can be accessed here:

http://www.biomedcentral.com/1471-2369/10/38/prepub
